# Supplementary material for: Application of 1-Hydroxy-4,5-Dimethyl-Imidazole 3-Oxide as Coformer in Formation of Pharmaceutical Cocrystals
Source: Pharmaceutics. 2020 Apr 15;12(4):359. doi: 10.3390/pharmaceutics12040359 (PMC7238160; doi:10.3390/pharmaceutics12040359)
Supplement: Supplementary file 1 [file pharmaceutics-12-00359-s001.pdf]

Supplementary Materials: Application of 1-Hydroxy-4,5-Dimethyl-Imidazole 3-Oxide as Coformer in Formation of Pharmaceutical Cocrystals

Aneta Wróblewska, Justyna Śniechowska, Sławomir Kaźmierski, Ewelina Wielgus, Grzegorz D. Bujacz, Grzegorz Mlostoń, Arkadiusz Chworos, Justyna Suwara and Marek J. Potrzebowski

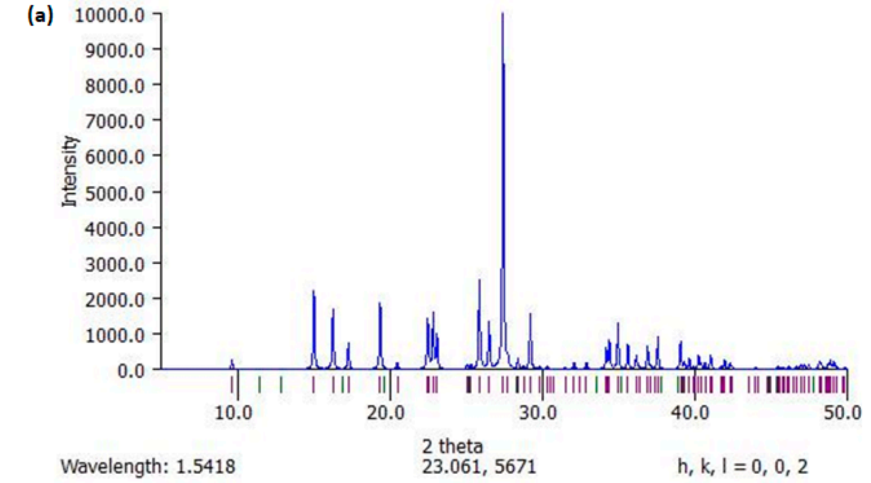

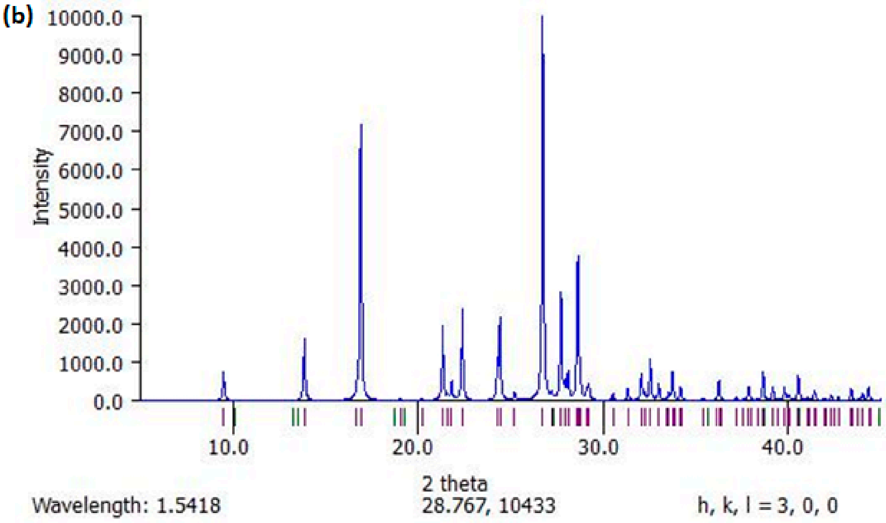

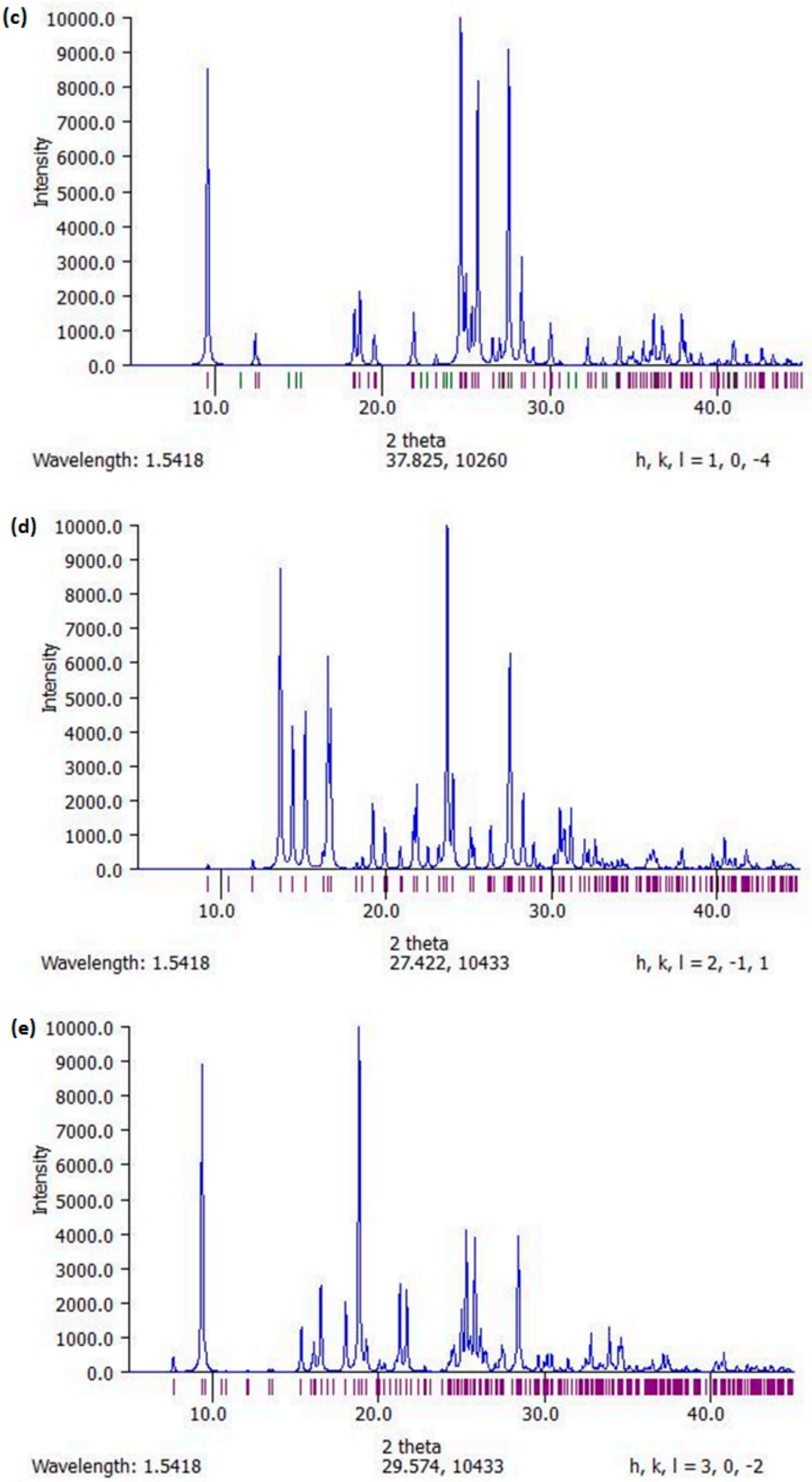

Figure S1. Calculated powder x-ray diffraction diffractograms (from top to bottom) TBA crystallized from acetonitryl (tautomer A)[1]; TBA crystallized from ethanol (tautomer B)[1]; TBA crystallized from water (tautomer B)[1]; HIMO; TBA/HIMO.

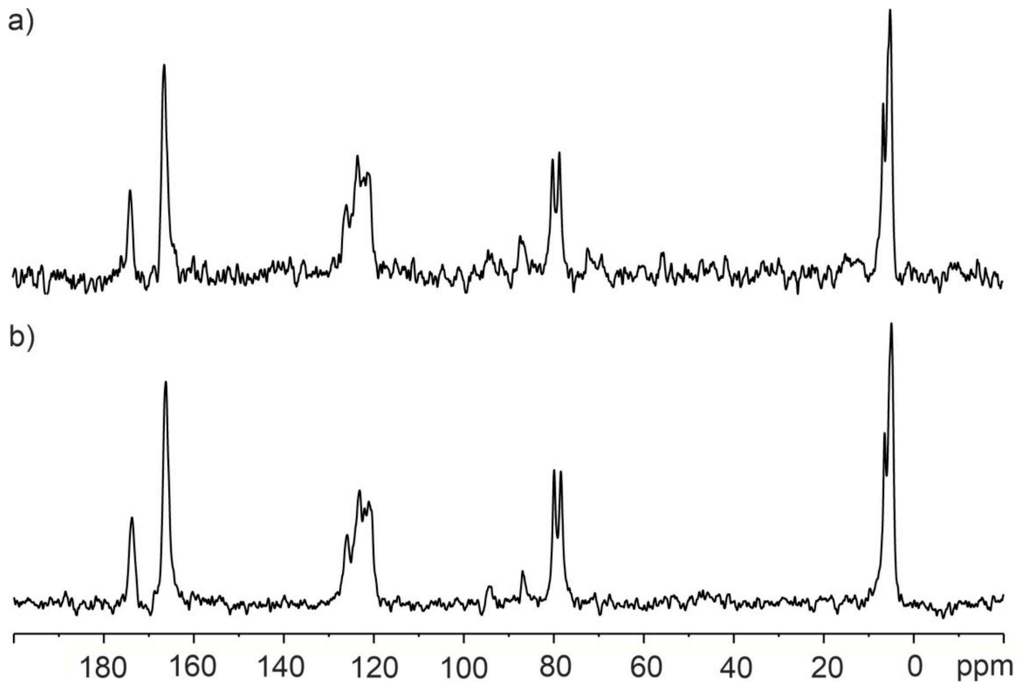

Figure S2. ^{13}C CP/MAS spectra recorded with a spinning rate of 8 kHz for TBA/HIMO cocrystals obtained by (**a**) grinding; (**b**) cocrystallization from MeOH.

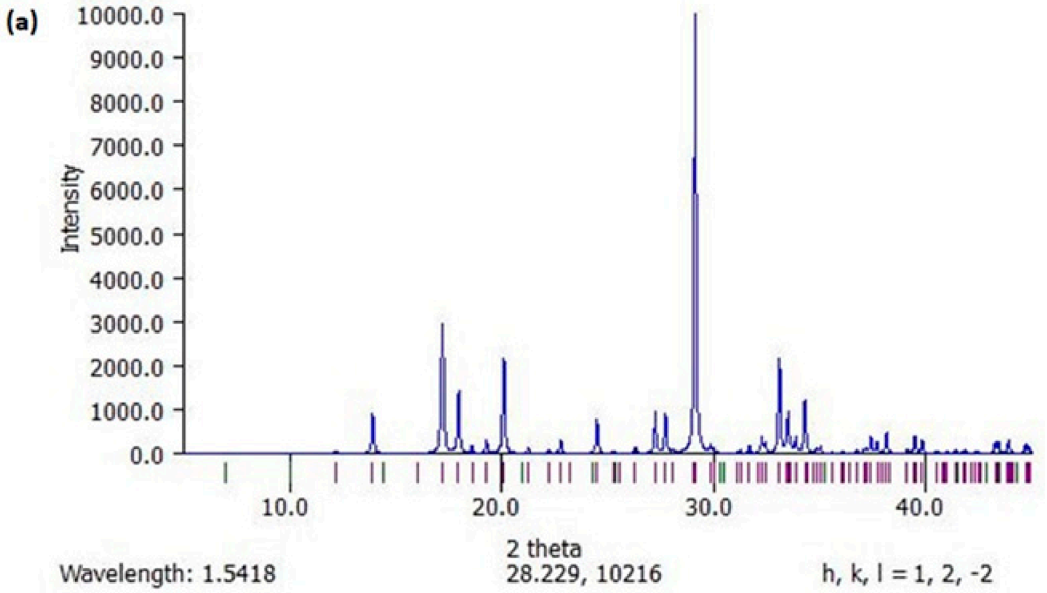

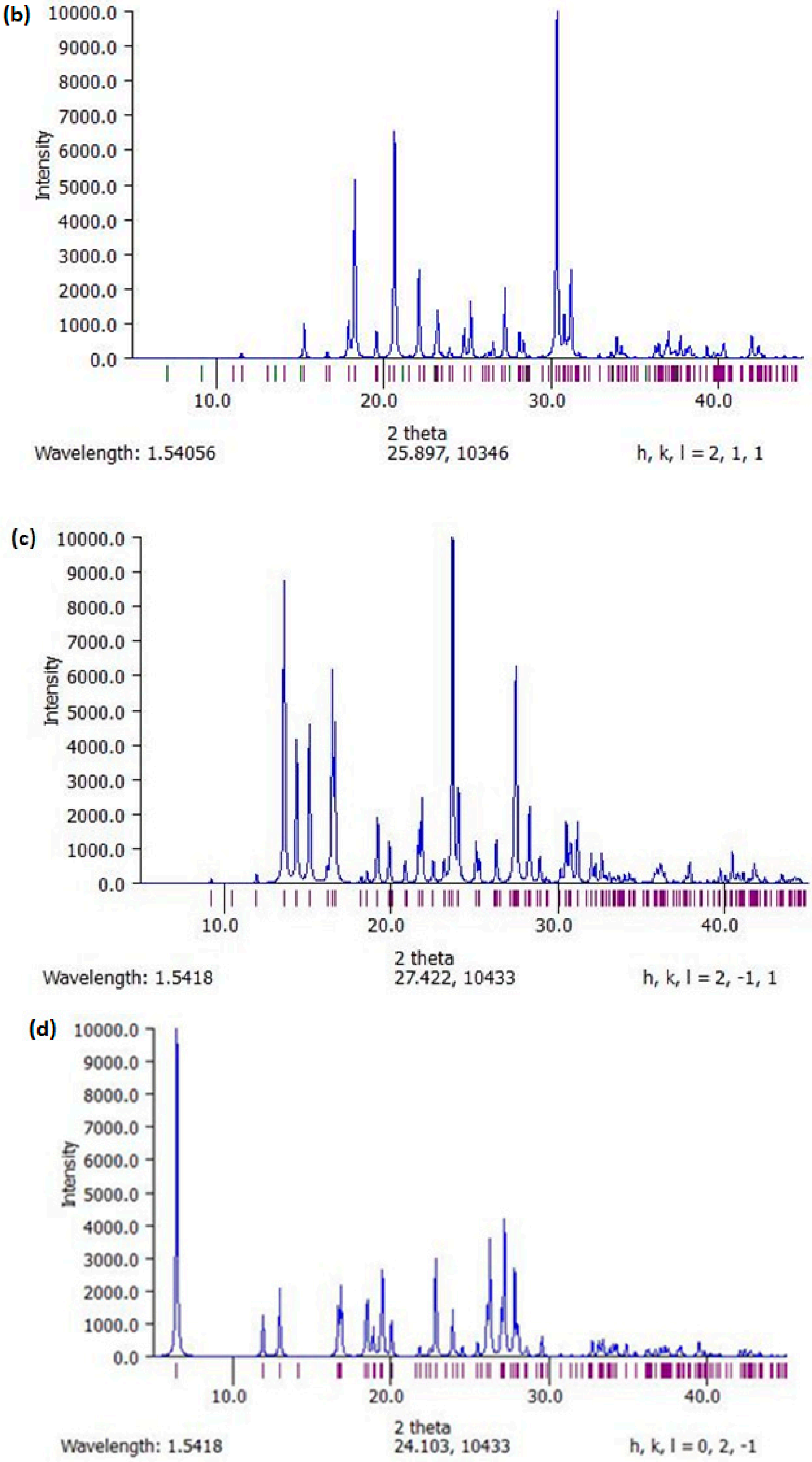
(b)
Intensity
2 theta
Wavelength: 1.54056
25.897, 10346
h, k, l = 2, 1, 1
(c)
Intensity
2 theta
Wavelength: 1.5418
27.422, 10433
h, k, l = 2, -1, 1
(d)
Intensity
2 theta
Wavelength: 1.5418
24.103, 10433
h, k, l = 0, 2, -1

Figure S3. Calculated powder x-ray diffraction diffractograms (from top to bottom) for; BA (form II)[2], BA dihydrate[3]; HIMO and BA/HIMO.

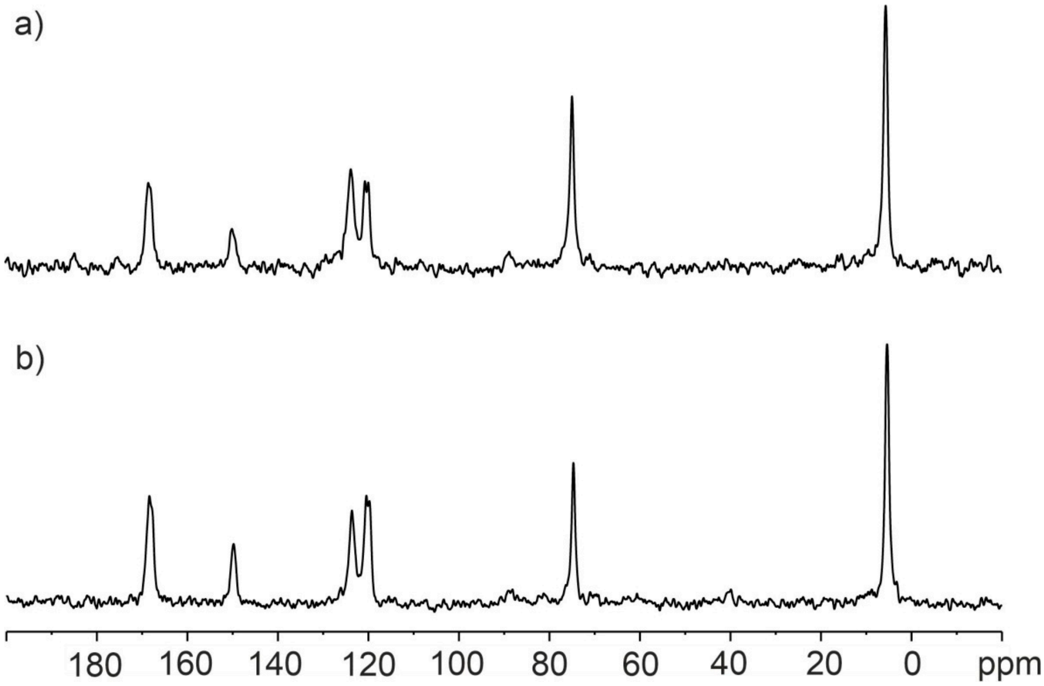

Figure S4. ^{13}C CP/MAS spectra recorded with a spinning rate of 8 kHz for BA/HIMO cocrystals obtained by (**a**) grinding; (**b**) cocrystallization from MeOH.

References

1. Chierotti, M.R.; Ferrero, L.; Garino, N.; Gobetto, R.; Pellegrino, L.; Braga, D.; Grepioni, F.; Maini, L. The Richest Collection of Tautomeric Polymorphs: The Case of 2-Thiobarbituric Acid. *Chem. Eur. J.* **2010**, *16*, 4347–4358.
2. Lewis T. C, Tocher D. A., Price S. L. An Experimental and Theoretical Search for Polymorphs of Barbituric Acid: The Challenges of Even Limited Conformational Flexibility Crystal Growth *&* Design **2004**, *4*, 979-987
3. Nichol G.S., Clegg W., *Acta Crystallographica,Section B: Structural Science*, A variable-temperature study of a phase transition in barbituric acid dihydrate **2005**, *61*, 464-472
